# Activity Clamp Provides Insights into Paradoxical Effects of the Anti-Seizure Drug Carbamazepine

**DOI:** 10.1523/JNEUROSCI.3697-16.2017

**Published:** 2017-05-31

**Authors:** Gareth Morris, Marco Leite, Dimitri M. Kullmann, Ivan Pavlov, Stephanie Schorge, Gabriele Lignani

**Affiliations:** Department of Clinical and Experimental Epilepsy, Institute of Neurology, University College London, WC1N 3BG London, United Kingdom

**Keywords:** carbamazepine, dynamic clamp, epilepsy, hippocampus, paradoxical effect, sodium channels

## Abstract

A major challenge in experimental epilepsy research is to reconcile the effects of anti-epileptic drugs (AEDs) on individual neurons with their network-level actions. Highlighting this difficulty, it is unclear why carbamazepine (CBZ), a frontline AED with a known molecular mechanism, has been reported to increase epileptiform activity in several clinical and experimental studies. We confirmed in an *in vitro* mouse model (in both sexes) that the frequency of interictal bursts increased after CBZ perfusion. To address the underlying mechanisms, we developed a method, activity clamp, to distinguish the response of individual neurons from network-level actions of CBZ. We first recorded barrages of synaptic conductances from neurons during epileptiform activity and then replayed them in pharmacologically isolated neurons under control conditions and in the presence of CBZ. CBZ consistently decreased the reliability of the second action potential in each burst of activity. Conventional current-clamp recordings using excitatory ramp or square-step current injections failed to reveal this effect. Network modeling showed that a CBZ-induced decrease of neuron recruitment during epileptic bursts can lead to an increase in burst frequency at the network level by reducing the refractoriness of excitatory transmission. By combining activity clamp with computer simulations, the present study provides a potential explanation for the paradoxical effects of CBZ on epileptiform activity.

**SIGNIFICANCE STATEMENT** The effects of anti-epileptic drugs on individual neurons are difficult to separate from their network-level actions. Although carbamazepine (CBZ) has a known anti-epileptic mechanism, paradoxically, it has also been reported to increase epileptiform activity in clinical and experimental studies. To investigate this paradox during realistic neuronal epileptiform activity, we developed a method, activity clamp, to distinguish the effects of CBZ on individual neurons from network-level actions. We demonstrate that CBZ consistently decreases the reliability of the second action potential in each burst of epileptiform activity. Network modeling shows that this effect on individual neuronal responses could explain the paradoxical effect of CBZ at the network level.

## Introduction

Progress in the pharmacological treatment of epilepsy has been hampered by the difficulty of linking the cellular effects of anti-epileptic drugs (AEDs) to their network actions. Epileptic seizures result from a complex interplay between multiple neuronal populations with differing intrinsic biophysical properties (Naze et al., 2015). Existing experimental techniques are limited in their ability to determine how AEDs change neuronal activity during a seizure because no two seizures are exactly the same and thus direct comparisons between baseline and AED conditions are impossible.

One of the most widely used AEDs for the treatment of partial-onset seizures is carbamazepine (CBZ) (Shorvon, 2000). The primary mechanism of action of CBZ, shared with phenytoin and lamotrigine, is use-dependent blockade of voltage-gated sodium channels (VGSCs) (McLean and Macdonald, 1986; Kuo, 1998; Vreugdenhil and Wadman, 1999; Mantegazza et al., 2010). Pyramidal neurons, rather than inhibitory interneurons, have been demonstrated to be the main targets of CBZ (Pothmann et al., 2014).

However, CBZ can become ineffective in patients with chronic epilepsy and, paradoxically, in some forms of epilepsy, CBZ can exacerbate seizures (Osorio et al., 2000; Remy et al., 2003; Gayatri and Livingston, 2006; Thomas et al., 2006; Gavrilovici et al., 2014). In some animal models of epilepsy, CBZ causes an increase in interictal spiking (Gigli and Gotman, 1991). In addition, *in vitro* models of epilepsy have revealed that CBZ can increase the frequency of epileptiform bursts (Quilichini et al., 2003). The mechanisms underlying these paradoxical effects of CBZ are unclear, although suggested mechanisms include changes in VGSC properties or network state-dependent changes in other membrane channels (Uebachs et al., 2010; Uebachs et al., 2012; Thomas and Petrou, 2013). In particular, bombardment of neurons with random suprathreshold synaptic inputs to model epileptic activity reveals that time spent at hyperpolarized potentials can change the effectiveness of AEDs significantly, potentially through modulation of calcium-activated potassium channels (Thomas and Petrou, 2013).

Here, we developed a new approach to isolate the cellular and network actions of CBZ: mimicking the excitatory and inhibitory synaptic inputs experienced by individual neurons during epileptiform activity using dynamic clamp (Chance et al., 2002; Prinz et al., 2004; Wolfart et al., 2005; Desai and Walcott, 2006; Piwkowska et al., 2008). This allowed us to compare the response of a neuron to the same inputs with and without CBZ, avoiding the confounding effect of changes in network state.

## Materials and Methods

### 

#### 

##### Animals and ethical approval.

All experimental procedures were performed in accordance with the UK Animals (Scientific Procedures) Act of 1986.

##### Slice preparation.

C57BL6 mice (RRID: IMSR_JAX:000664) of either sex (2–4 weeks old) were anesthetized with isofluorane (∼3% in oxygen, 2 L/min) and killed by cervical dislocation. Brains were quickly dissected into ice-cold oxygenated slicing solution containing the following (in mm): 205 sucrose, 2.5 KCl, 26 NaHCO_3_, 1.2 NaH_2_PO_4_·H_2_O, 10 glucose, 5 MgCl_2_, and 0.1 CaCl_2_ and cut into 300 μm horizontal slices using a 7000 SMZ vibratome (Campden Instruments) to give access to the ventral hippocampus. Slices were stored submerged in oxygenated recording artificial CSF (aCSF) containing the following (in mm): 10 glucose, 125 NaCl, 3 KCl, 26 NaHCO_3_, 1 MgCl_2_, 1.25 NaH_2_PO_4_·H_2_O, and 2 CaCl_2_ at room temperature for at least 1 h before recording.

##### High potassium *in vitro* epilepsy model.

For *in vitro* recording of epileptiform activity, slices were transferred to a “membrane chamber” (Hill and Greenfield, 2011; Morris et al., 2016) to permit simultaneous patch-clamp and local field potential (LFP) recordings of epileptiform activity. A fresh membrane insert was prepared at the start of each experimental day. All recordings were made using an AxoClamp 700B (Molecular Devices), filtered at 6 kHz, digitized at 10 kHz with an NI-6221 board (National Instruments), and recorded using WinEDR software (John Dempster, University of Strathclyde, Glasgow, UK). For LFP recordings, a glass micropipette (∼3–4 MΩ) was filled with recording aCSF and placed into hippocampal CA1 stratum pyramidale. Patch-clamp recordings from nearby cells were made using glass micropipettes (∼4–5 MΩ) filled with internal solution containing the following (in mm): 135 K-gluconate, 4 KCl, 10 HEPES, 4 Mg-ATP, 0.3 Na-GTP, and 10 Na_2_ phosphocreatine. All recordings had access resistance <20 MΩ; current-clamp recordings were rejected if action potentials did not overshoot 0 mV and voltage-clamp recordings were rejected if the holding current exceeded 100 pA. To determine pyramidal neuron firing patterns during epileptiform activity, action potentials were recorded in the cell-attached configuration using a micropipette filled with standard aCSF. All recordings were performed at 32°C and with a perfusion rate of ∼16 ml/min.

Epileptiform activity was induced by increasing the potassium concentration in the aCSF to 10 mm. In all experiments, 50 μm DL-(−)-2-amino-5-phosphonopentanoic acid (DL-APV, Abcam) was added after the onset of epileptiform activity to isolate AMPAR-mediated from NMDAR-mediated excitatory currents. Where relevant, an identical aCSF containing 30 μm CBZ in DMSO (Sigma-Aldrich) was applied to the slice.

##### Activity clamp.

To create a template trace for activity clamp, we simultaneously recorded from two neighboring CA1 pyramidal neurons in the voltage-clamp configuration during epileptiform activity. One neuron was held at −80 mV and one at 0 mV to record the temporal profiles of AMPA and GABA_A_ receptor-mediated currents, respectively. The holding potentials of the two neurons were swapped during recording to ensure that synaptic inputs during epileptiform discharges were qualitatively similar in both cells. The slight contamination of the excitatory template recorded at −80 mV by GABAergic conductances (in this case, producing inward currents) could result in an overestimation of the excitatory conductances in our templates by ∼9%. However, we found that, whereas the E/I balance and the time course of conductances varied considerably between individual bursts, the effects of CBZ were independent of these parameters. Therefore, this is unlikely to be a significant confounder in our experiments.

A conductance profile was generated for each current according to: *g* = *I*/(*V*_rev_ − *V*_m_) where *g* is the calculated conductance, *I* is the recorded current, and *V*_rev_ and *V*_m_ are the reversal and membrane potentials, respectively. Considering the liquid junction potential (LJP, 13.6 mV), the estimated driving force was 93.6 mV for excitatory and −41.4 mV for inhibitory currents, taking into account the shift in GABA_A_ equilibrium potential due to the high extracellular potassium concentration. Dynamic clamp software (Signal 6.0; Cambridge Electronic Design) and a Power3 1401 (Cambridge Electronic Design) were used to inject both excitatory and inhibitory conductance templates simultaneously in a neuron recorded in current-clamp configuration (iteration frequency 15 kHz). *E*_rev_ was set to 0 mV and −75 mV for excitatory and inhibitory conductances, respectively, and corrected for an LJP of 14.9 mV. Miniature Excitatory postsynaptic currents (EPSCs) were recorded from CA1 neurons in the presence of tetrodotoxin (1 μm), DL-APV (50 μm), and picrotoxin (PTX, 30 μm) to determine the kinetics of unitary AMPAR-mediated synaptic currents for the excitatory synaptic conductance template. Incremental synaptic conductances were injected in recorded neurons to establish the conductance threshold for AP generation.

##### Current-clamp recordings.

Current-clamp recordings for activity clamp were performed in standard external solution (see slice preparation section above) in the presence of DL-AP5 (50 μm), CNQX (10 μm), and PTX (30 μm) to block the NMDA, AMPA/kainate, and GABA_A_ receptors, respectively. The internal solution contained the following (in mm): 126 K gluconate, 4 NaCl, 1 MgSO_4_, 0.02 CaCl_2_, 0.1 BAPTA, 15 glucose, 5 HEPES, 3 ATP, and 0.1 GTP, pH 7.2 with KOH. Neurons with holding current >100 pA and *R*_a_ >20 MΩ upon whole-cell break-in in voltage-clamp mode and membrane potential less negative than −60 mV in current clamp were not considered for analysis. A 1440 Digidata (Molecular Devices) or Power3 1401 (Cambridge Electronic Design) interface and Multiclamp 700A (Molecular Devices) amplifier were used for all of the activity-clamp recordings and the associated current-clamp recordings. The sampling frequency was set to 10 kHz for the current-clamp recording in accordance with the activity-clamp template sampling rate.

##### Data analysis.

Dynamic clamp data and the associated current-clamp data were analyzed with custom MATLAB (The MathWorks, RRID:SCR_001622) scripts. Action potentials were accepted for analysis if they crossed 0 mV and the maximal derivative of their rising slope exceeded 10 mV/ms. Action potential timings refer to the voltage threshold (the first point at which dV/dt > 10 mV/ms) of the waveform.

##### Network modeling.

We used a refractory exponential integrate and fire (rEIF) neuron model (Badel et al., 2008) to simulate the single neurons of an excitatory population. Each neuron was described by the following differential equation:

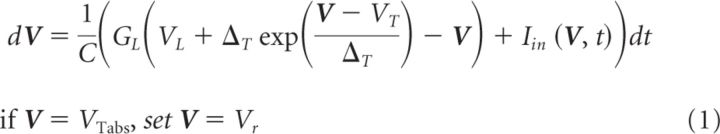
 Here each neuron is described by a state space variable *V*, corresponding to its membrane voltage; an external input current *I_in_*(*V*, *t*), where the static parameter *C* is the membrane capacitance, *V*_Tabs_ is an absolute spike threshold, and *V_r_* is the reset voltage after the spike. There are also four time-varying parameters corresponding to the leak conductance *G_L,_* he leak reversal potential at rest *V_L_*, the spike width parameter Δ*_T_*, and the soft spike threshold *V_T_*, which are described by the following exponential relaxation equations reflecting the spike refractory characteristics:

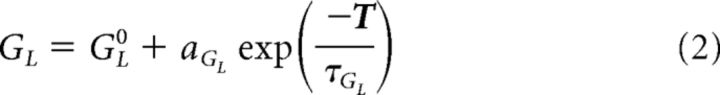


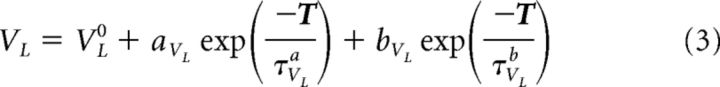


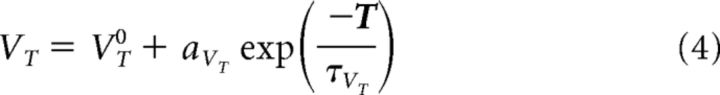


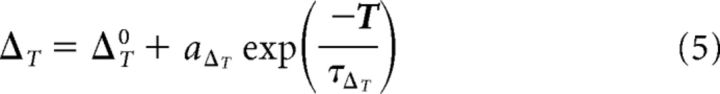
 where *G*_*L*_^0^, *V*_*L*_^0^, *V*_*T*_^0^, and Δ_*T*_^0^ are the basal values of the parameters; *a_G_L__*, *a_V_L__*, *b_V_L__*, *a_V_T__*, and *a*_Δ*_T_*_ are the deviation from the basal values due to spiking; and τ*_G_L__*, τ*_V_L__^a^*, τ*_V_L__^b^*, τ*_V_T__*, and τ_Δ*_T_*_ are exponential decay time constants. Here we also introduce the state variable ***T*** corresponding to the time since the neuron last spiked.

The parameters used for the simulations were based on results described previously (Badel et al., 2008) for pyramidal neurons and fine-tuned manually so that the model fit the behavior of a sample neuron under the dynamic clamp control condition for a typical burst input. To model the effect of CBZ at the single neuron level, we increased the value of τ*_V_T__* such that the spiking of the model neuron matched the measurements under the CBZ dynamic clamp condition. This increase can be interpreted to translate phenomenologically the slower recovery time of sodium channels from inactivation after spiking due to CBZ. To simulate the dynamic clamp condition the input current function, *I_in_*(***V***, *t*), was set to the following:

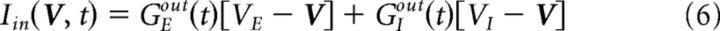
 where *G_E_^out^* and *G_I_^out^* are the excitatory and inhibitory conductance templates, respectively, and *V_E_* and *V_I_* are the excitatory and inhibitory reversal potentials, respectively.

Once the single neuron parameters were tuned, we built a network consisting of 100 model neurons connected all-to-all via excitatory conductance-based synapses. For each of the 10 rounds of simulation, the connection weights were randomly drawn from a uniform distribution, *U*, between 0 and 1 and are represented by the matrix Γ*_E_* as follows:

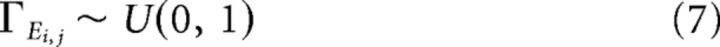
 To model the bursting behavior, we followed the method of Staley et al. (1998), in which the bursting activity ceases because of exhaustion of a releasable pool of presynaptic glutamate vesicles. In our model, each time a neuron spikes, a fixed fraction of its available vesicles is released and the rate of replenishment of the available pool of glutamate vesicles is proportional to the number of free vesicle sites available (Staley et al., 1998). This is summarized by the following differential equation:

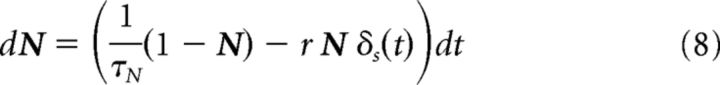
 where ***N*** is the normalized number of glutamate vesicles available, *r* is the ratio of vesicles released per spike, τ*_N_* is the vesicle recovery time constant, and δ*_s_*(*t*) represents a train of Dirac delta impulse functions at the times when the neuron spikes (conforming to jumps in the ***N*** variable). The efferent excitatory conductance per spike that a neuron elicits in the network is then assumed to be proportional to the number of glutamate vesicles released at the time of spiking and to follow a first-order exponential decay as follows:

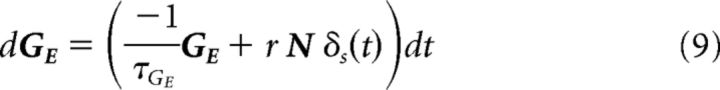
 where *G*_E_ is the normalized postsynaptic excitatory conductance and τ*_G_E__* is the time constant of its exponential decay. The total synaptic conductances that the neurons receive are then linear combinations of these, with the resulting current input function described by the following:

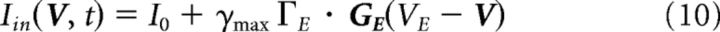
 where *I*_0_ is a tonic current input to all neurons and γ_max_ is a scaling factor on the connectivity matrix Γ*_E_*. In this instance, we need to acknowledge explicitly that the boldfaced state variables, in the case of network simulations, represent all neurons of the population in vector form. The parameters τ_N_, *r*, *I*_0_, and γ_max_ were set such that the network for the control condition would show bursts qualitatively similar to the ones observed *in vitro* (Table 1). To simulate the effect of CBZ in the network simulations, the only change in the model parameters was the same change in τ*_V_T__*, as in the single neuron simulations.

**Table 1.** Computational model parameters

*C*	170 pF	*a_V_L__*	16 mV	*CBZ* τ*_V_T__*	15 ms	*r*	0.3
*V_Tabs_*	−37 mV	τ_V_L__^a^	25 ms	Δ_T_^0^	2 mV	τ*_G_E__*	10 ms
*V_r_*	−43 mV	*b_V_L__*	−10 mV	*a*_Δ_*T*__	0 mV	*I*_0_	128 pA
*G*_*L*_^0^	6.8 nS	τ_*V*_*L*__^b^	100 ms	τ_Δ_*T*__	0.005 ms	σ	170 mV/s^1/2^
*a_G_L__*	9 nS	*V*_*T*_^0^	−52 mV	*V_E_*	0 mV	γ_*max*_	267 nS
τ*_G_L__*	30 ms	*a_V_T__*	15 mV	*V_I_*	−56 mV		
*V*_*L*_^0^	−75 mV	*ctrl* τ*_V_T__*	13 ms	τ_*N*_	8 s		

For completeness, the state variable representing the time since last spike can be described by the following equation:

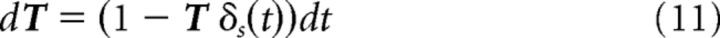
 Finally, we add a small stochastic term to the membrane voltage of each neuron, σ*dW*, representing heterogeneity on their inputs. To solve the resulting system of ensuing stochastic differential equations, we use the Euler–Maruyama numerical method with a step size of 0.1 ms. The MATLAB code implementing the simulations will be made publically available at github.com/mfpleite/rEIF-burst-CBZ.

##### Experimental design and statistical analysis.

All of the experimental data were obtained in hippocampal slices from at least three mice, which is consistent with the sample sizes used in the field; both sexes were represented. Individual statistical analyses and details of experimental design are described in detail alongside the each experiment in the Results section. Deviation from normal distributions was assessed using D'Agostino–Pearson's test, and the *F* test was used to compare variances between two sample groups. Paired Student's two-tailed *t* test or Wilcoxon matched-pairs signed-rank test were used as appropriate to compare means. To compare two groups at different time points, we used two-way ANOVA, followed by Bonferroni *post hoc* test for functional analysis. Statistical analysis was performed using Prism (GraphPad Software, RRID:SCR_002798) and SPSS (RRID:SCR_002865).

## Results

### CBZ increases epileptiform activity in slices exposed to high potassium

**Figure 1.**
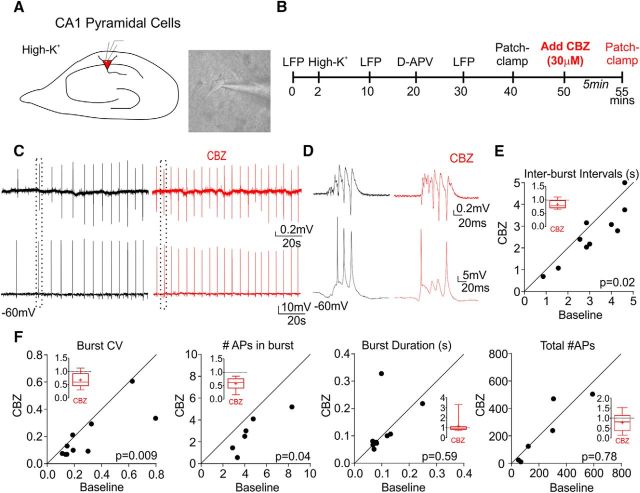
Paradoxical effect of CBZ in the high K^+^ model of epileptiform activity. ***A***, Representative image of the patch-clamp approach. ***B***, Experimental timeline. ***C***, Simultaneous LFP (top trace) and current-clamp (bottom trace) recordings in high K^+^ and DL-APV before (black) and after (red) 5 min of 30 μm CBZ application. ***D***, Expanded view of the traces from the dotted square in ***C*** showing bursts of APs (bottom) during LFP epileptiform discharges (top). ***E***, Scatter plots of the interburst intervals. ***F***, Scatter plots of coefficient of variation of the interburst intervals, number of APs in each burst, burst duration, and total number of APs calculated over 2 min before and after CBZ (paired Student's *t* test or Wilcoxon matched-pairs signed-rank test for nonparametric values). Inset panels show summary bar plot after CBZ with *y*-axes normalized to baseline. *n* = 10 slices from 4 mice.

LFP activity was recorded simultaneously with the firing activity of single pyramidal neurons in hippocampal CA1 (Fig. 1). Epileptiform activity was elicited by 10 mm K^+^ and consisted of brief LFP transients, during which pyramidal neurons fired short bursts of action potentials (Fig. 1*C*,*D*). Blocking NMDA receptors with 50 μm DL-APV had little effect on bursting. In the presence of 30 μm CBZ, the frequency of epileptiform discharges increased (Fig. 1*E*, *t*_(9)_ = 2.70, *p* = 0.02 Student's *t* test) and became more regular, reflected by a decrease in the coefficient of variation (CV, SD/mean) of interburst intervals (Fig. 1*F*, *p* = 0.009, Wilcoxon matched-pairs test). CBZ thus paradoxically increases epileptiform activity in the high K^+^ model, consistent with previous reports of CBZ-induced increases in epileptiform bursting in the low-magnesium model (Uebachs et al., 2010) or in kindled cat amygdala (Gigli and Gotman, 1991). In contrast to this effect of CBZ on the population bursting activity, we observed a consistent decrease in the number of action potentials in each burst (Fig. 1*F*, *p* = 0.04, Wilcoxon matched-pairs test). However, this was not associated with changes in the duration of bursts (*t*_(8)_ = 0.55, *p* = 0.59, paired Student's *t* test) and the overall number of APs over time (2 min) remained constant (*p* = 0.78, Wilcoxon matched-pairs test) before and after application of CBZ (Fig. 1*F*, for burst CV and duration, *n* = 10 slices from 4 mice; for AP counts, *n* = 6 cells, 6 slices, and 3 mice).

### Creating a template for comparison of epileptiform conductances

**Figure 2.**
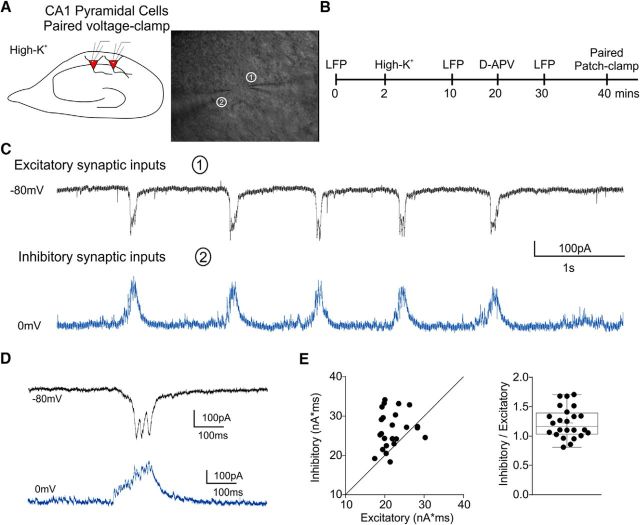
Recording of the activity-clamp template during epileptiform activity. ***A***, ***B***, Graphical representation of the experimental plan. Dual patch-clamp recordings of neighboring CA1 pyramidal neurons were obtained in high K^+^ solution (***A***). Before dual whole-cell path clamp, LFP was recorded to verify the presence of epileptiform activity. DL-APV was applied to exclude NMDAR-mediated component of excitatory currents and to allow simultaneous recording of excitatory and inhibitory currents (***B***). ***C***, Simultaneous recording of excitatory (AMPA, black trace, −80 mV holding potential) and inhibitory (GABA_A_, blue trace, 0 mV holding potential) synaptic inputs in the CA1 pyramidal neurons. ***D***, Expanded view of the synaptic currents recorded during an individual burst. ***E***, Scatter plot showing the relationships between inhibitory and excitatory charges (left) and their ratios (right) showing variability among individual bursts.

**Figure 3.**
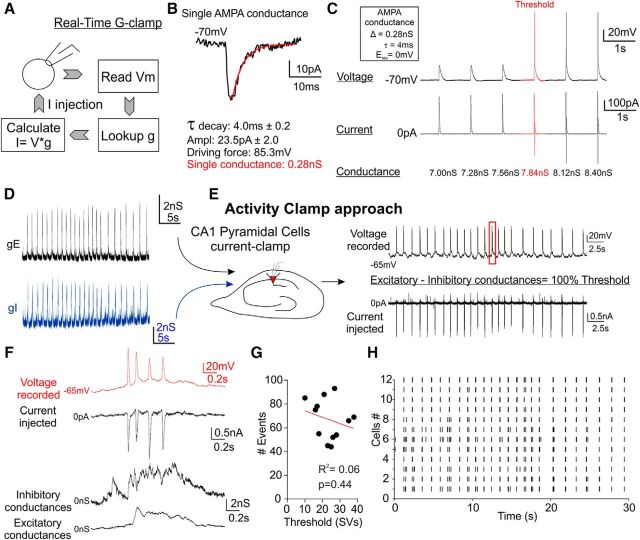
Activity-clamp template preparation and modeling epileptiform activity in single neurons with activity clamp. ***A***, Activity-clamp cycle based on the real-time conductance clamp of both excitatory and inhibitory epileptiform conductances. The dynamic clamp software reads *V*_m_ from the neuron (top), acquires the synaptic conductance (*g*_syn_) from the activity-clamp template (right), calculates current to be injected from *I* = *V*_m_ * *g*_syn_ and injects the appropriate synaptic current to the neuron in current-clamp configuration. ***B***, ***C***, Calibration of activity-clamp amplitude. ***B***, Miniature EPSCs were recorded in the presence of tetrodotoxin from CA1 pyramidal cells to create a unitary AMPAR-mediated synaptic conductance template (*n* = 9 cells from 3 animals). ***C***, Magnitude of unitary AMPA conductance was incremented to define the conductance threshold for AP generation to scale the activity-clamp template for each recording. ***D***, Excitatory (*g*_E_) and inhibitory (*g*_I_) conductance templates derived separately from recorded AMPAR- and GABA_A_R-mediated currents divided by the corresponding calculated driving force (excitatory: LJP 13.6 mV; holding −80 mV; *V*_rev_ 0 mV; driving force: 93.6 mV; inhibitory: LJP 13.6 mV; holding 0 mV; *V*_rev_ −55 mV; driving force −41.4 mV). ***E***, Graphical representation of the activity-clamp approach. *g*_E_ and *g*_I_ templates were injected simultaneously (*g*_syn_) to mimic synaptic input impinging onto a single neuron during epileptiform activity. Conductance templates were scaled to the maximal *g*_E_ matched neuronal conductance threshold (i.e., the minimum number of single AMPA conductances need to trigger an AP) established at the start of a recording. ***F***, Burst induced by current injection using both conductance templates simultaneously. ***G***, Plot of the AP numbers versus the conductance threshold shows no relationship between these two parameters (*n* = 12; Pearson correlation coefficient; *p* = 0.44). ***H***, Raster plot of all APs in the entire activity-clamp protocol for every cell recorded (*n* = 12).

Do CBZ-induced changes in spiking during bursts reflect altered network activity or alterations in the spiking dynamics at the single-cell level? To isolate the effects of CBZ on single-cell firing during epileptiform discharges from other effects of the drug that potentially affect network behavior, we developed a method to mimic synaptic barrages received by neurons during network bursts. Two neighboring CA1 pyramidal neurons were patched simultaneously in voltage-clamp mode during epileptiform bursts in 10 mm K^+^. By holding the two neurons at different membrane potentials, GABA_A_ inhibitory currents were recorded in one and AMPA excitatory currents were recorded in the other (Fig. 2). The resulting recordings consisted of 24 consecutive excitatory and inhibitory current profiles, each of which represented a single epileptiform burst. The ratio of inhibitory to excitatory currents varied from burst to burst (Fig. 2*E*). The excitatory and inhibitory current traces were then converted into conductance templates that were subsequently applied to pharmacologically isolated neurons using dynamic clamp (Fig. 3*A*,*D*; see Materials and Methods).

To reduce variability among neurons due to differences in intrinsic excitability, the excitatory conductance threshold for action potential generation was established for each neuron and the conductance templates were scaled according to this threshold. To provide a biophysically meaningful scaling parameter, we estimated the minimal number of unitary synaptic AMPA conductances required to elicit an AP. Averaged amplitude and kinetics of miniature AMPA postsynaptic currents recorded from CA1 pyramidal neurons (Fig. 3*B*, *n* = 9 cells from 3 mice) were used to create a unitary AMPAR synaptic conductance waveform, which was applied to neurons while incrementing its amplitude until an AP was triggered (Fig. 3*C*).

### Reproducing epileptiform activity in single neurons with activity clamp

We next recorded from CA1 neurons in the presence of ionotropic (NMDA, AMPA/kainate, and GABA_A_) receptor blockers and applied the scaled conductance template to simulate the simultaneous presence of both excitatory and inhibitory epileptiform synaptic conductances. Injecting the combined templates to CA1 neurons held in current clamp at −70 mV evoked burst activity similar to that recorded in the high K^+^ model (Fig. 3*D–F*). The number of APs in each burst varied among neurons and among individual bursts in the template, highlighting two sources of heterogeneity in neuronal responses during epileptiform discharges (Fig. 3*G*,*H*).

### Activity clamp distinguishes effects of CBZ at the single-cell level from network actions

**Figure 4.**
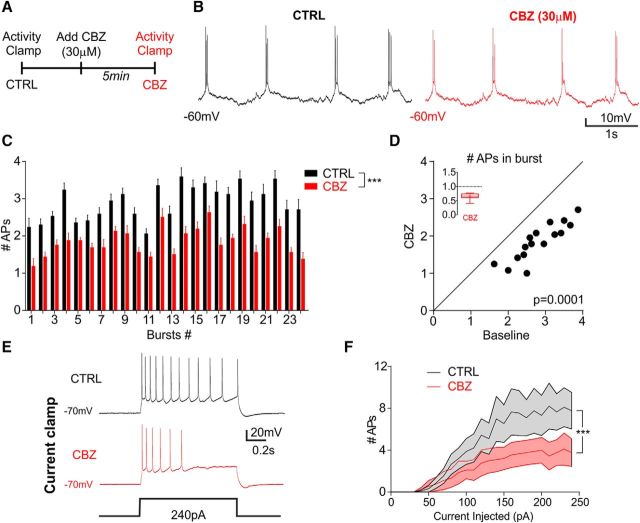
Activity clamp reinforces the CBZ-induced decrease in number of APs in epileptiform bursts. ***A***, Experimental design showing sequential delivery of activity-clamp traces containing conductances from identical epileptiform bursts in the absence and presence of CBZ. ***B***, Neuronal voltage response to the same activity-clamp template before and after application of 30 μm CBZ. ***C***, Number of APs in each burst triggered by activity clamp before and after addition of CBZ (*n* = 16 from 4 animals, *p < 0.001; multiple *t* tests corrected for multiple comparison with the Holm–Sidak method). ***D***, Scatter plot of the average number of APs in bursts calculated for 24 bursts before and after CBZ (*n* = 16 from 4 animals, ***p = 0.0001, paired Student's *t* test). Inset panels show summary bar plot after CBZ with *y*-axes normalized to baseline. ***E***, Representative traces showing the response to individual bursts in current-clamp recordings before and after CBZ application. ***F***, Number of APs versus current injected before and after CBZ (*n* = 9 from 3 animals; ***p < 0.001, two-way ANOVA followed by Bonferroni correction for multiple comparisons). Data are shown as mean ± SEM.

To permit paired comparisons, activity clamp was performed on the same neurons before and after 5 min perfusion with 30 μm CBZ (Fig. 4*A*,*B*). The conductance threshold before and after CBZ did not change significantly (control 20.17 ± 2.29 nS; CBZ 23 ± 2.99 nS; mean ± SEM, *n* = 16 cells from 4 mice, *t*_(11)_ = 2.06, *p* = 0.064, paired Student's *t* test). Despite the heterogeneity of bursts, repeated stimulation with activity clamp revealed that treatment with CBZ consistently caused a decrease in number of APs during each burst (Fig. 4*C,D*; C: control vs CBZ *n* = 16 cells from 4 mice, all *p* < 0.001, multiple *t* tests corrected for multiple comparison with the Holm–Sidak method; Fig. 4*D*: control vs CBZ *n* = 16 cells from 4 mice, *t*_(15)_ = 12.56, *p* = 0.0001, paired Student's *t* test). This result was similar to the effect of CBZ on the number of APs induced by square pulse current injection protocols (Pothmann et al., 2014) (Fig. 4*E*,*F*; *n* = 9 cells from 3 mice; *F*_(1,8)_ = 14.74, *p* = 0.005, two-way repeated-measures ANOVA followed by Bonferroni *post hoc* test) and by high K^+^ perfusion (Fig. 1*D*,*E*).

**Figure 5.**
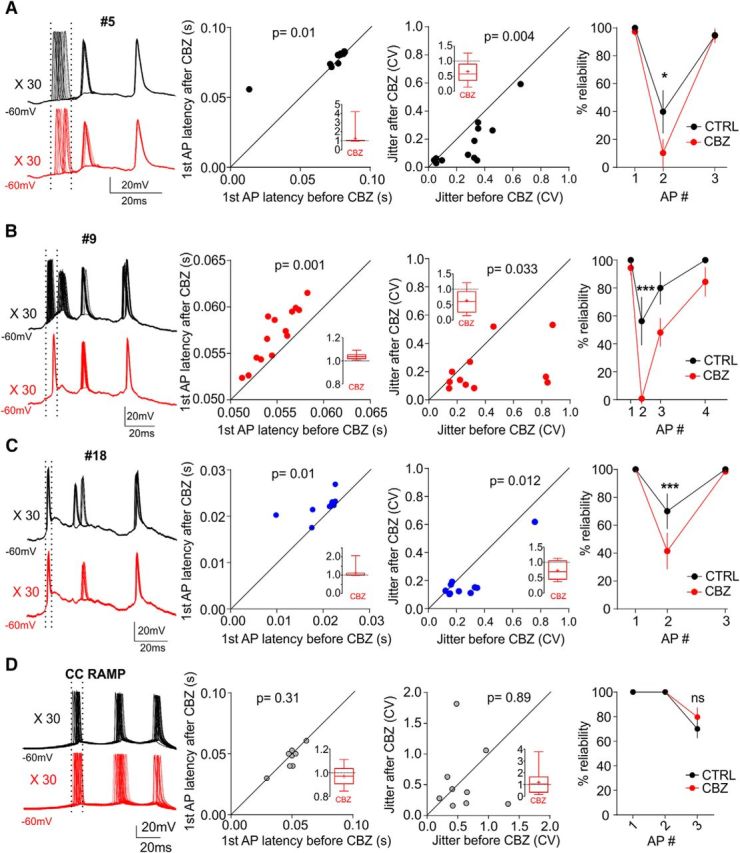
CBZ delayed the first AP, reduced its variability, and decreased the reliability of the second AP in activity-clamp induced epileptiform bursts. ***A***–***D***, left: Superimposed traces of bursts #5 (***A***), #9 (***B***), and #18 (***C***) and in the ramp protocol (***D***) for one representative neuron repeated 30 times before (black trace) and after (red trace) CBZ application. Middle left, Scatter plot of the first AP latency before and after CBZ in 3 random bursts (black = burst #5, red = burst #9, and blue = burst #18) and in the current-clamp ramp for each cell (*n* = 12 from 3 animals, ramp *n* = 9 from 3 animals). The latency in activity clamp is defined from the start of the rising phase of excitatory conductance for each burst. The latency in the ramp is defined from the beginning of the ramp pulse. Paired Student's *t* test or Wilcoxon matched-pairs signed rank test for nonparametric values were used. Middle right, Scatter plot of the jitter, represented as CV, before and after CBZ in 3 random bursts (black is burst #5, red is burst #9, and blue is burst #18) and in the current-clamp ramp for each cell (*n* = 12 from 3 animals, ramp *n* = 9 from 3 animals). Data are shown as individual neurons. Paired Student's *t* test. Right, AP reliability in bursts #5 (***A***), #9 (***B***), and #18 (***C***) and in the ramp protocol (***D***). Data are shown as mean ± SEM (*n* = 12 from 3 animals, ramp *n* = 9 from 3 animals). Two-way ANOVA followed by correction for multiple comparisons was used. Inset panels show summary bar plot after CBZ with *y*-axes normalized to baseline.

In longer trains of activity, the reduction in AP number has been attributed to increasing activity of calcium-dependent potassium channels (Thomas and Petrou, 2013). Our work reveals an additional effect occurring early in a burst even if neurons fire just a few APs. To quantify this, we picked three random bursts with different E/I ratios from the activity-clamp protocol to compare AP timing and reliability (Fig. 5*A–C*) with and without CBZ. For comparison, we measured the same parameters in each neuron using a ramp current protocol (Fig. 5*D*). The ramp amplitude was set as the minimal current needed to elicit at least 3 APs in a 100 ms ramp (range 150–250 pA). Activity clamp revealed that the first AP timing was consistently delayed by CBZ in all bursts (Fig. 5*A–C*). This delay was not apparent when the ramp protocol was used (Fig. 5*A–C*: Wilcoxon matched-pairs signed rank test for activity clamp; burst #5, *p* = 0.01; burst #9, *p* = 0.001; burst #18, *p* = 0.01′ *n* = 12 cells from 3 mice; Fig. 5*D*: paired Student's *t* test for current clamp, *t*_(8)_ = 1.078, *p* = 0.31; *n* = 9 cells from 3 mice). Analysis of the CV revealed a significant decrease in the variability of first AP timing in the presence of CBZ (before vs after CBZ, Fig. 5*A–C*; burst #5, *t*_(11)_ = 3.611, *p* = 0.004; burst #9, *t*_(10)_ = 2.456, *p* = 0.033; burst #18, *t*_(10)_ = 3.084, *p* = 0.012, paired Student's *t* test; *n* = 10–12 cells from 3 mice). Again, this effect was not observed in response to the excitatory current ramp (Fig. 5*D*, *t*_(8)_ = 0.13, *p* = 0.89, paired Student's *t* test, *n* = 9 cells from 3 mice). In addition to delaying the first AP in the burst and increasing its precision in response to synaptic input, CBZ also reduced the reliability of the second AP in each burst (Fig. 5*A–C*, two-way ANOVA followed by Bonferroni *post hoc* test; burst #5, *t*_(11)_ = 3.085, *p* < 0.05; burst #9, *t*_(24)_ = 4.51, *p* < 0.001; burst #18, *t*_(16)_ = 5.388, *p* < 0.001; *n* = 12 cells from 3 mice). This effect was not observed in response to the ramp protocol (Fig. 5*D*, two-way ANOVA followed by Bonferroni *post hoc* test, *t*_(16)_ = 1.92, *p* > 0.05; *n* = 9 cells from 3 mice). Among the possible explanations for the decrease of the second AP reliability are the delay of the first AP and binding to sodium channels in inactivated states after the first AP.

**Figure 6.**
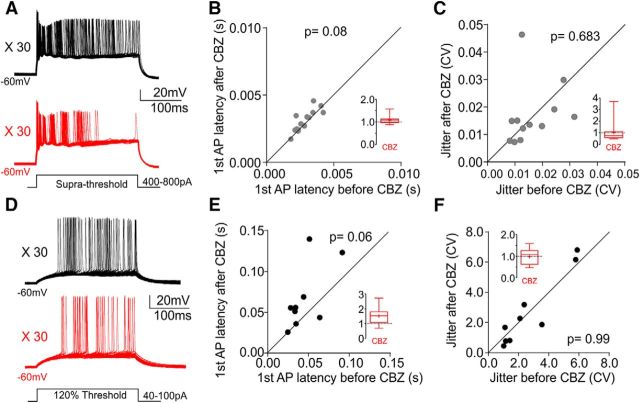
Square pulse current injection protocols do not reveal the effect of CBZ observed in activity-clamp experiments. ***A***, Representative traces of current-clamp recordings of 30 repeated current steps. The amplitude of the suprathreshold current step was set as the maximal current reached in activity-clamp bursts for each cell. The black trace represents before CBZ and the red trace after CBZ. ***B***, Scatter plot of the first AP latency before and after CBZ for each cell (*n* = 12 from 3 animals). The latency is defined from the beginning of the square pulse. Data are shown as individual neurons. Paired Student's *t* test. ***C***, Scatter plot of the jitter, represented as CV, before and after CBZ in the current-clamp square pulse for each cell (*n* = 12 from 3 animals). Data are shown as individual paired neurons. Wilcoxon matched-pairs signed rank test for nonparametric values. ***D***, Representative traces of current-clamp recordings of 30 repeated current steps. The amplitude of the current step was set as 120% of the current threshold need to elicit an AP with 250 ms square step pulses for each cell. The black trace represents before CBZ and the red trace after CBZ. ***E***, Scatter plot of the first AP latency before and after CBZ for each cell (*n* = 9). The latency is defined from the beginning of the square pulse. Data are shown as individual neurons. Paired Student's *t* test was used. ***F***, Scatter plot of the jitter, represented as CV, before and after CBZ in the current-clamp square pulse for each cell (*n* = 9 from 3 animals). Data are shown as single paired neurons. Wilcoxon matched-pairs signed-rank test for nonparametric values was used. Inset panels show summary bar plot after CBZ with *y*-axes normalized to baseline.

Can other conventional current-clamp protocols used to evoke neuronal spiking reveal CBZ effects that occur early in a burst? To address this question, we used a suprathreshold square pulse repeated 30 times (Fig. 6*A*). The amplitude of the square pulse was set as the maximal current reached in the same cell during activity-clamp bursts. The first AP delay and the decrease in CV produced by CBZ application in activity-clamp experiments were not apparent in this protocol (Fig. 6*B*,*C*; AP latency, *t*_(14)_ = 1.948, *p* = 0.08; jitter, *t*_(11)_ = 0.42, *p* = 0.683; paired Student's *t* tests). This may have been due to the steep onset of the current step tightly controlling the timing of the first AP. To allow more variability, we next injected lower-amplitude current steps (Fig. 6*D*, 120% of current threshold). This protocol showed a slight increase in the delay of the first AP (Fig. 6*E*, *t*_(8)_ = 2.178, *p* = 0.06, paired Student's *t* test), but no differences were observed in the CV (Fig. 6*F*, *t*_(8)_ = 0.11, *p* = 0.99, paired Student's *t* test). Unlike the ramp protocol, both current injections induced firing with almost an order of magnitude difference in spiking precision. Furthermore, action potentials elicited by these current-clamp protocols did not follow stereotyped temporal patterns, thus not allowing the analysis of reliability of the second AP.

Activity clamp thus reveals activity-dependent effects of AEDs on individual neurons that are not seen using conventional electrophysiological recording techniques.

### Network modeling reconciles CBZ effects on single neurons with its action at a network level

**Figure 7.**
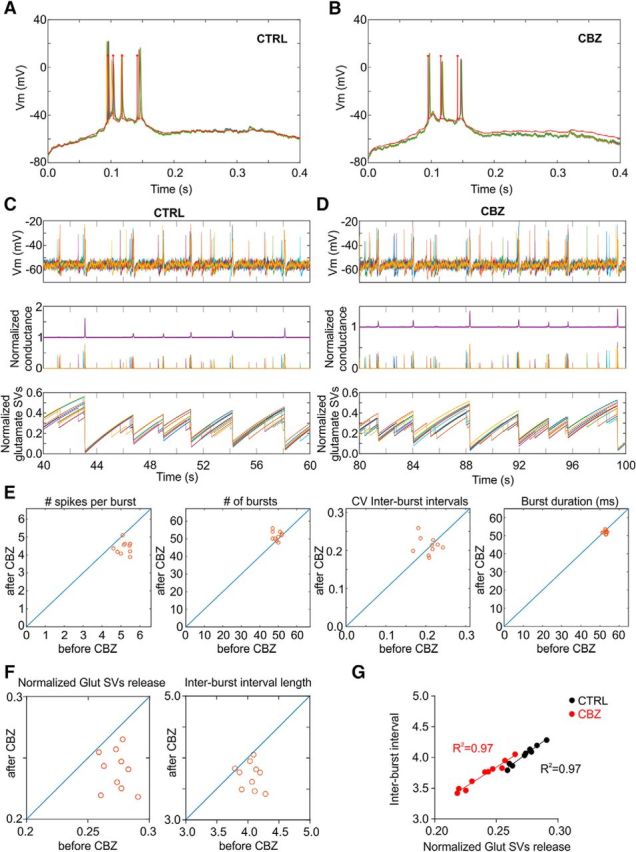
Network modeling reconciles the effects of CBZ effects on single neurons with its action at a network level. ***A***, Membrane voltage for activity clamp burst #9 in control conditions for sample *in vitro* runs (green) and computational model fit (red). ***B***, Same as ***A***, but in CBZ conditions (increased τ*_V_T__* value). ***C***, Traces for 10 sample model neurons during a 20 s window illustrating the network model bursting behavior. Top, Membrane voltages for each neuron. Middle, Efferent normalized conductance outputs for each neuron (thin lines) and average normalized conductance output of the whole population (thick purple line, with DC offset added for improved visualization). Bottom, Normalized number of glutamate vesicles available over time for each neuron. ***D***, Same as ***C*** but in CBZ conditions. ***E***, Summary of 10 network simulations with randomly sampled connectivity matrices Γ*_E_* in 200-s-long periods in control versus CBZ conditions. ***F***, Normalized glutamate SV release during simulated bursts and the interburst interval length of 10 network simulations with randomly sampled connectivity matrices Γ*_E_* in 200-s-long periods in control versus CBZ conditions. ***G***, Correlation between average normalized glutamate SV release during simulated bursts and the following interburst interval of 10 network simulations with and without CBZ.

How does the apparent inhibitory action of CBZ at the single neuron level result in the increased frequency and regularity of network bursting? The link between single neuron activity and network dynamics cannot be investigated with activity clamp. Therefore, to address this, we used a computational approach recreating the changes in single neuronal dynamics caused by CBZ (Fig. 7). First, we used a phenomenological point neuron model, the rEIF model (Badel et al., 2008), to capture the single neuron membrane voltage and spiking dynamics seen during activity clamp. We fine-tuned the parameters of the model to reproduce the membrane voltage traces recorded in the control condition (Fig. 7*A*). We then parsimoniously captured the effect of CBZ by changing the time constant with which the spike threshold relaxes to the baseline value in the relative refractory period after each spike (τ*_V_T__*) (Vreugdenhil and Wadman, 1999). An increase in this parameter led to the absence of the second action potential, as observed *in vitro* (Fig. 7*B*).

To link these intraburst changes with the altered frequency of bursts, we explored the effects of the single-cell changes on network output. In particular, we investigated whether the depletion of glutamate vesicles during rapid activity, as proposed in an earlier study of network bursting, can explain an increased burst frequency in response to fewer APs during individual intracellular bursts (Staley et al., 1998; Staley et al., 2001; de la Prida et al., 2006; Jones et al., 2007). We used 10 rounds of simulations with randomly selected synaptic weights using the established fine-tuned neuronal parameters with precise bursting mechanisms. Because glutamate vesicles are replenished after a burst, the network excitability increases (Staley et al., 1998). This means that each random spike from the neurons in the network elicits larger and larger excitatory conductances in their postsynaptic counterparts as vesicles are replenished (Fig. 7*C*,*D*). Once the amount of excitation in the network reaches a critical value, the network becomes prone to burst. The number of spikes emitted during each burst dictates the number of glutamate vesicles depleted from each neuron and thus would be expected to affect the time that they take to recover (Fig. 7*C*,*D*).

We simulated 200 s with the control parameters and then changed only τ*_V_T__* to simulate the presence of CBZ using the same network structures. In the conditions in which a single parameter was changed to mimic CBZ, the number of APs per burst was reliably smaller than in control conditions. Indeed, we found that reduced number of spikes in the burst allowed the network to trigger a subsequent burst sooner, thus increasing the overall frequency of discharges (Fig. 7*E*,*F*). Importantly, the correlation between glutamate release and the next interburst interval was unaltered before and after CBZ, suggesting that CBZ does not alter the bursting properties of the network by other mechanisms (Fig. 7*G*).

These results illustrate a plausible mechanism whereby the effects of CBZ at the single neuron level, as unveiled by the activity-clamp protocol, are sufficient to explain the paradoxical increase in burst frequency at the network level.

## Discussion

The mechanisms underlying the paradoxical effects of AEDs are not completely understood. New insights into the mechanisms relating the actions of AEDs at the cellular and network levels could improve the pharmacological treatment of epilepsy. The activity-clamp strategy developed here provides an opportunity for distinguishing changes in neuronal response to synaptic inputs from changes in population network activity. By allowing repeated delivery of identical epileptiform inputs, activity clamp also allows direct and detailed comparison of how different treatments alter neuronal bursting behavior.

Although some studies have shown that that paradoxical effects of CBZ may be ascribed to alteration of VGSC properties and to the network state (Uebachs et al., 2010; Uebachs et al., 2012; Thomas and Petrou, 2013), CBZ mainly reduces excitatory neurotransmission by acting on pyramidal neurons without affecting feedback and feedforward inhibition (Pothmann et al., 2014; Paz and Huguenard, 2015). However, CBZ can also exacerbate seizures. Here, we combined dynamic clamp and a computational approach to uncover a candidate mechanism of the paradoxical effect of CBZ. We have reproduced realistic synaptic conductances observed during epileptiform activity (activity clamp) by simulating the presence of identical input barrages in different neurons. This allowed us to compare neuronal behavior in response to complex synaptic inputs in the presence of CBZ. Our recordings revealed that the effects of CBZ in bursts lead to reduced reliability of the second AP and consequently fewer APs in response to a given synaptic burst input. The effect on the second AP could be associated with the delayed generation of the first AP observed in activity-clamp recordings. The effects of CBZ have been shown to depend on the network state (Thomas and Petrou, 2013) and the realistic voltage trajectory obtained in activity clamp can isolate the steady-state effects of CBZ, from the dynamic effects that can occur during bursts of epileptiform activity.

Are the effects on APs revealed here related to paradoxical effects of CBZ sometimes observed in clinical practice? The action of CBZ has been studied previously using conventional whole-cell electrophysiological protocols and *in silico* modeling (McLean and Macdonald, 1986; Vreugdenhil and Wadman, 1999; Uebachs et al., 2010; Thomas and Petrou, 2013; Gavrilovici et al., 2014; Pothmann et al., 2014). The activity-clamp data uncovered a decreased reliability of the second AP and thus fewer APs in each burst. The computer simulations suggest that this is consistent with depletion of the releasable pool of glutamatergic vesicles determining the interburst interval (Staley et al., 1998; Staley et al., 2001; de la Prida et al., 2006; Jones et al., 2007). Bursts with fewer APs have less glutamate release per burst, reducing the time taken to replenish vesicles to a sufficient level to facilitate a subsequent burst (Staley et al., 1998; Staley et al., 2001; de la Prida et al., 2006; Jones et al., 2007). It is possible that the effects of CBZ on the timing of the first AP in a burst and the reduction in second AP reliability both contribute to the pro-epileptic increase in epileptiform network activity that we observed in slices treated with CBZ. Indeed, the increased first AP reliability in bursts in the presence of CBZ uncovered by activity clamp may be associated with the increase in synchronicity that we and others observed at the network level (Fig. 1) (Mainen and Sejnowski, 1995; Buzsáki and Draguhn, 2004). Altered network synchronization is one of the features that can regulate the dynamics of epileptic events (Jiruska et al., 2013). Spiking reliability is strongly correlated with the amplitude of stimulus fluctuations (Mainen and Sejnowski, 1995). Therefore, by mimicking barrages of synaptic inputs precisely during epileptiform activity, activity clamp provides a better means for detection of subtle but physiologically more relevant adjustments in spike reliability than conventional current-clamp protocols.

The correlation between interictal events and ictogenesis in humans is still under debate (Avoli et al., 2006; Karoly et al., 2016). One hypothesis is that increased neural excitability promotes epileptic spikes, which may reach a critical spatial or temporal density and lead to seizures (Jensen and Yaari, 1988; Staley et al., 2011; Chauvière et al., 2012); another suggests that spike rate is largely unchanged or even reduced before seizures (de Curtis and Avanzini, 2001; Avoli et al., 2006; Karoly et al., 2016). Although the possibility that the observed increase of epileptiform bursts (analogous to interictal activity in humans) could be related to a decrease in ictogenesis (i.e., an antiepileptic effect) cannot be excluded, further *in vivo* studies will need to be performed to test this possibility. However, even if the increase in epileptiform events protected against ictogenesis, these data provide new insights into the mechanism of action of AEDs.

Here, we showed another possible explanation for the paradoxical action of CBZ. We demonstrated that AEDs such as CBZ, the mechanism of which is the use-dependent blockade of voltage-gated sodium channels, can decrease AP reliability in individual neurons within bursting circuits, which in turn can increase network activity by reducing the refractoriness of bursts. Our data, combined with previous results showing that the action of CBZ is predominantly on pyramidal neurons (Pothmann et al., 2014) and depends on the network state (Thomas and Petrou, 2013) and that interictal bursts in the hippocampus are regulated by presynaptic glutamate release and replenishment (Staley et al., 1998; Staley et al., 2001; de la Prida et al., 2006), provide an important mechanistic understanding of the action that is overlooked by conventional electrophysiological approaches.

More broadly, the dynamic-clamp approach allows one to analyze differences in spike timing and precision resulting from altered synaptic inputs. Because conductance templates could be created for any pattern of (patho)physiological brain activity that can be captured in single-cell recordings, the technique could be widely applicable in other fields. The method has some limitations. First, variability between different neuronal populations means that individual templates for each cell type need to be obtained. Here, we focused on pyramidal neurons, but further work would be needed to compare interneurons, which are comparatively sparse and represent a highly heterogeneous population (Klausberger and Somogyi, 2008). Ideally, templates would be captured *in vivo*; however, the voltage-clamp recordings at glutamate and GABA reversal potentials are difficult to obtain *in vivo* during epileptiform activity. A further limitation of the activity-clamp approach is that the inhibitory and excitatory conductances are both applied via the same somatic recording pipette, which fails to reflect subcellular segregation of synaptic inputs (Kohus et al., 2016).

Activity clamp permits comparisons between responses to the same bursts in the same neurons in varying experimental conditions. By removing two sources of variability, we were able to determine a previously unknown effect of CBZ, which provides a mechanistic explanation for its paradoxical effect at the network level.
